# Modeling Spectroscopic Properties of Ni^2+^ Ions in the Haldane Gap System Y_2_BaNiO_5_

**DOI:** 10.1007/s00723-013-0448-8

**Published:** 2013-03-31

**Authors:** C. Rudowicz, P. Gnutek, S. Kimura, M. Açıkgöz, Y. Y. Yeung

**Affiliations:** 1 Modeling in Spectroscopy Group, Institute of Physics, West Pomeranian University of Technology Szczecin, Al. Piastów 17, 70-310 Szczecin, Poland; 2 Institute for Materials Research, Tohoku University, Katahira 2-1-1, Sendai, 980-8577 Japan; 3 Faculty of Art and Sciences, Bahcesehir University, Besiktas, Istanbul, Turkey; 4 Department of Science and Environmental Studies, Hong Kong Institute of Education, Hong Kong SAR, People’s Republic of China

## Abstract

Modeling of spin Hamiltonian parameters enables correlation of crystallographic, spectroscopic, and magnetic data for transition ions in crystals. In this paper, based on the crystallographic data and utilizing the point-charge model and superposition model, the crystal field parameters (CFPs) are estimated for Ni^2+^(3*d* ^8^) ions in the Haldane gap system Y_2_BaNiO_5_. The CFPs serve as input for the perturbation theory expressions and the crystal field analysis package for microscopic spin Hamiltonian modeling of the zero-field splitting parameters (ZFSPs) *D* and *E*. Results of an extensive literature search of the pertinent crystallographic data, experimental ZFSPs, and model parameters are briefly outlined. The modeling aims at verification of the experimental ‘single ion anisotropy’ parameters and explanation of the controversy concerning the maximal rhombic distortion |*E*/*D*| ≈1/3 reported for Ni^2+^ ions in Y_2_BaNiO_5_. The preliminary results call for reanalysis of some magnetic studies of the Haldane gap systems.

## Introduction

The Haldane gap systems exhibit several key features [1–5], namely, (1) integer spin (*S* = 1, 2) on the transition ions, (2) one-dimensional anisotropic antiferromagnetic (AF) chains, (3) no long range magnetic ordering observed down to very low *T*; (4) a nonmagnetic “spin-liquid” ground state (singlet) separated from a branch of triplet excitations by a finite energy gap Δ, so-called Haldane gap, conjectured by Haldane in 1983 [6]. Existence of the Haldane gap in the energy spectrum has profound implications for magnetic and spectroscopic properties of these systems. Importantly, no such gap exists for the half-integer spin systems. Numerous theoretical and experimental studies, including inelastic neutron scattering (INS), magnetization, high-magnetic field and high-frequency electron magnetic resonance (HMF–EMR), have been carried out to confirm Haldane’s conjecture [1–5]. Most of the Haldane gap systems discovered so far [1–5] are based on Ni^2+^ (*S* = 1) ions and exhibit either orthorhombic (e.g., Y_2_BaNiO_5_ [YBNO], Ni(C_2_H_8_N_2_)_2_NO_2_(ClO_4_) [NENP], Ni(C_3_H_10_N_2_)_2_NO_2_(ClO_4_) [NINO], Ni(C_5_D_14_N_2_)_2_N_3_(PF_6_) [NDMAP], Ni(C_5_D_14_N_2_)_2_N_3_(ClO_4_) [NDMAZ]) or axial site symmetry (CsNiCl_3_, Ni(en)_3_(ClO_4_)_2_·H_2_O [NEN3P], [Ni(C_2_H_8_N_2_)_2_NO_2_](BF_4_) [NENB], Ni(C_2_H_8_N_2_)_2_NO_2_(PF_6_) [NENF], Ni_2_V_2_O_7_, Ni(C_3_H_10_N_2_)_2_N_3_(ClO_4_) [NINAZ], PbNi_2_V_2_O_8_, (CH_3_)_4_NNi(NO_2_)_3_ [TMNIN]). In addition, a Haldane gap system based on V^3+^ (*S* = 1) ions AgVP_2_S_6_ is known, see, e.g., Ref. [7].

In view of the scientific merit of Haldane gap studies [1–5], an extensive literature search has recently been carried out, which has revealed several controversies and inconsistencies. It has also turned out that interpretation of raw experimental data using various theoretical methods may yield disparate values of the zero-field splitting parameters (ZFSPs) for single Ni^2+^ ions. To solve the pertinent problems, we have embarked on a project aimed at modeling of the ZFSPs *D* and *E* for single Ni^2+^ ions in various Haldane gap systems. Importantly, modeling of ZFSPs, which enables correlation of crystallographic, spectroscopic, and magnetic data for transition ions in crystals, may provide a better insight into properties of these systems. The modeling aims at verification of the single ion anisotropy data and explanation of the controversy concerning the maximal rhombicity ratio |*E*/*D*| ≈ 1/3 reported by some authors for YBNO [8–10], which contradicts the first INS results [11] indicating a large axial *D* value with the *E* term not considered. Original motivation for this study was to solve this controversy. Major focus of this paper is on YBNO, whereas to a lesser extent also NENP is discussed. The preliminary results call for re-analysis of some magnetic studies of the Haldane gap systems.

## General Aspects Concerning Haldane Gap Systems

For the integer spin *S* = 1 systems described by a spin Hamiltonian that includes only Heisenberg exchange interactions (*J*) within one-dimensional (1D) antiferromagnetic (AF) chains and weak interchain interactions (*J′*) one Haldane gap Δ is predicted [6]. Since the Haldane gap originates as a solution of the Hamiltonian for the whole 1D AF chain [6, 12, 13], the gap is due to quantum mechanical and many-body effects [1–5]. Taking into account also the so-called ‘single-ion anisotropy’ (SIA) terms [1–5] (in fact, as explained below, the ZFS terms) yields two and three gaps for systems with axial and orthorhombic site symmetry [12, 13], respectively. An illustrative energy-level scheme for NENP, which exhibits orthorhombic site symmetry, may be found in Fig. 4 of Lu et al. [14] for the case with the magnetic field **B**∥*b* axis. The sequence of the excited states, which depends on the sign of the respective axial ZFSP, is well represented therein.

Interpretation of the effective energy levels of the Haldane gap spin *S* = 1 systems and the nature of the Haldane energy gap(s) between the ground singlet (*S*_T_ = 0) state and the excited triplet (*S*_T_ = 1) states is still somewhat confused in literature. Consequently, the distinction between the well-studied ZFS within the single Ni^2+^ (*S* = 1) ion states in crystals, see, e.g., Refs. [15–19], and the ZFS within the excited *S*_T_ = 1 states of a Haldane gap spin *S* = 1 system, see, e.g., Refs. [1–14], is not clear in some papers. To avoid confusion, we shall describe the former ZFS by the parameters *D*, *E*, whereas the latter ZFS by the parameters D*, E*. The cases of inadvertent misinterpretation of the two types of ZFSPs by some authors will be discussed in the full paper. Another common misinterpretation occurring in the literature concerns the mechanism of the splitting of the excited triplet (*S*_T_ = 1) states. The statements like *‘*SIA splits the excited *S*_T_ = 1 states’ are not true, since this splitting is due to a combined effect of the Heisenberg exchange interactions and the ZFS (alias ‘SIA’) of single Ni^2+^ ions [12, 13].

The terminology used in Haldane gap studies [1–5] requires clarification. In the magnetism literature, the SIA, or equivalently the ‘magnetocrystalline anisotropy’ (MCA), is considered as a special case of the ‘magnetic anisotropy’ (MA), see, e.g., references in the reviews [20, 21] and the book [22]. In general, the magnetic anisotropy may be also due to the anisotropic exchange interactions. The so called ‘SIA’ terms [1–14] represent, in fact, the zero-field splitting (ZFS), or equivalently the fine structure, terms [23, 24] for the single Ni^2+^ (*S* = 1) ion. Hence, such terminology constitutes the MA = ZFS confusion [20, 21] consisting in referring to a quantity related actually to the notion ‘ZFS’ using incorrectly the name of another well-defined notion, i.e., SIA (MCA). The true magnetic anisotropy, regardless of its origin, is defined [22] as the part of the free energy of the crystal depending on the direction of the magnetization *M* in crystal. It is described in terms of the functions of the direction cosines of *M* and the magnetic anisotropy constants {*K*_*i*_}, which depend on the physical parameters, including the single-ion ZFSPs and the exchange constants. Hence, the actual magnetic anisotropy constants {*K*_*i*_} are physically distinct quantities than the single-ion ZFSPs, i.e., (*D*, *E*)—in the conventional notation or $B_{ 2}^{q}$ (or $b_{ 2}^{q}$) in the extended Stevens operators notation, which is now prevailing in EMR and magnetism studies [23, 24]. Hence, the parameters *D*, *E* [1–5], most often named in Haldane gap studies [1–5] as the ‘SIA’ parameters are actually the single-ion ZFS parameters and not the ‘SIA’ ones. The two types of quantities SIA (or equivalently MA, MCA) and ZFS should not be identified each with the other.

## Analysis of Experimental Data and Theoretical Models

The spin Hamiltonian (SH) used in studies of Haldane gap *S* = 1 systems, besides the Heisenberg exchange interactions, includes the ‘SIA’ terms (which, for the reasons given above, should be referred to as the ‘ZFS’ terms) usually given in the form [1–5]:

1 $$ H_{\text{ZFS}} = DS_{z}^{2} + E\left( {S_{x}^{2} - S_{y}^{2} } \right),$$

that is, with the constant term: [−*DS*(*S* + 1)/3] omitted [23, 24]. Note that the invalid truncated forms of the second-rank orthorhombic spin Hamiltonians have also been employed in the studies of Haldane gap antiferromagnets as reviewed in Ref. [25].

The abbreviations adopted below for experimental methods are: MS = magnetic susceptibility, χ(*T*), and/or magnetization *M*(*T*), INS = inelastic neutron scattering, whereas for the type of sample: P = powder, SC = single crystal, PC = polycrystalline. The temperature ranges at which the experimental measurements were done are also indicated. Symbols for the Haldane gaps are: Δ_0_ is the value of the gap in absence of ‘SIA’, Δ_*p*_ is the two (three) Haldane gaps for systems with axial (orthorhombic) site symmetry [1–5]; the subscripts *p* used in the source papers are retained. Below the available experimental data on YBNO and theoretical models used for their interpretation are summarized and analyzed. The values of ZFSPs and other relevant quantities listed in the source papers in the units of (K) or (meV) were converted to (cm^−1^) to facilitate direct comparison. Additional comments on aspects bearing on the reliability of the results and their interpretation are also provided in brief.

Darriet and Regnault [11]: YBNO sample: P; MS: 1.8–300 K; INS: 32 and 117 K; SH: equivalent to Eq. (1) with the first term named ‘SIA’ and *E* ≡ 0. From analysis of INS data at low temperature they obtained: Δ_0_ = 89 ± 6, Δ_*z*_ ≈ 129 ± 8, Δ_*xy*_ ≈ 69 ± 4. Interpretation of Δ_*i*_ was based on the theoretical expressions adapted from Refs. [12, 26]:

2 $$ \Updelta_{xy} \approx \Updelta_{0} - \alpha_{xy} D;\quad \Updelta_{z} \approx \Updelta_{0} + \alpha_{\text{z}} D $$

with the coefficients α_*xy*_ and α_*z*_ arising from numerical solutions of the Heisenberg antiferromagnetic chain of spin *S* = 1 with nearest-neighbor exchange interaction *J* and ‘easy-plane SIA’ *D* [12]. The values α_*xy*_ ≈ 0.57 and α_*z*_ ≈ 1.37 were used in Ref. [11]. Note that α_*z*_ ≈ 1.37 differs slightly from 1.41 in Ref. [12], whereas no α_*i*_ has been found in Ref. [26]. The average gap was calculated by an approximate relation:

3 $$ \Updelta_{0} \approx ( 2\Updelta_{xy} + \Updelta_{z} )/ 3 $$

Using Eqs. (2) and (3), the ZFSP for the single Ni^2+^ (*S* = 1) ion was obtained [11] as *D* = 32 ± 7 cm^−1^, whereas the *E* term was not considered.

The question of interpretation of the ‘average’ gap ∆_0_ and the other two Haldane gaps ∆_*p*_ in terms of appropriate energy levels as well as the directional properties of the gaps ∆_*p*_, implied by the subscripts *p* = *z* and *xy*, will be dealt with in the full paper. Here we only note that such notation resembles the correspondence between the spin *S* = 1 states (of any nature) denoted in the Cartesian coordinates as $\left| {1, \, M_{\text{s}} = \pm 1 > \sim } \right|xy >$ and $\left| {1, \, M_{\text{s}} = 0 > \sim } \right|z >$. The notation [11] differs from the more appropriate one used by Golinelli et al. [12], which avoids ascribing any directional property to the gaps, instead, the gaps are defined in Ref. [12] in terms of the energy transitions between the ground singlet (*S*_T_ = 0) state and the excited triplet (*S*_T_ = 1) states as *G* ^(−)^ = *E*(*S*_T_ = 0, *S* ^z^ = 0) → *E*(*S*_T_ = 1, *S* ^z^ = ±1) and *G* ^(+)^ = *E*(*S*_T_ = 0, *S* ^z^ = 0) → *E*(*S*_T_ = 1, *S* ^z^ = 0). Note that the sign of *D*(*S*_T_ = 1) ≡ D* appears to be negative judging from Δ_*xy*_ ≈ 8.5 ± 0.5 (*S*_T_ = 1, *M*_s_ = ±1), Δ_*z*_ ≈ 16 ± 1 (*S*_T_ = 1, *M*_s_ = 0).

Xu et al. [9]: YBNO sample: two SCs; INS: 10 K measured in the three axes: (*x*, *y*, *z*) = (*c*, *b*, *a*); SH: equivalent to Eq. (1) with the ZFS terms named ‘anisotropy terms’. From global fit of INS data, they obtained the energies of the three modes as Δ_*a*_ = 62.1 (8), Δ_*b*_ = 71.0 (8), Δ_*c*_ = 79.0 (8); similar values were obtained from infinite chain length gap fit. Interpretation of Δ_*i*_ was based on the theoretical relations adapted from Refs. [12, 27]:

4 $$ \Updelta_{ \bot } = \Updelta_{0} - 0. 5 7D\quad \Updelta_{\parallel } = \Updelta_{0} + 1. 4 1D. $$

The average gap Δ_av_ ≈ 69.4(8) was calculated by an approximate relation:

5 $$ \Updelta_{\text{av}} = (\Updelta_{a} + \Updelta_{b} + \Updelta_{c} )/ 3. $$

The derived values of the ZFSPs for the single Ni^2+^ (*S* = 1) ion are [9]: *D* ≈ −6.5, *E* ≈ 2.0 (cm^−1^).

Doubts arise concerning the reliability of the ZFSP values reported in Ref. [9]. Equation (4) applies only for systems with axial [12]. For systems with orthorhombic site, symmetry equations involving three gaps [13] should be used. Actually, *D* was obtained in Ref. [9] by taking ‘Δ_⊥_ to be the average of the transverse mode energies in Y_2_BaNiO_5_’, which yielded ‘Δ_0_ ≈ 8.6 meV and *D* ≈ −0.81 meV (easy-axis anisotropy)’, i.e., *D* ≈ −6.5 cm^−1^. Since no *E*-term is considered in Eq. (4), the value of *E* was obtained in Ref. [9] indirectly from the relation stated as: ‘For *D*/*J* = 0.18 the splitting of the transverse modes equals 4*E*’ [27]. This yielded the estimated value of *E* in Y_2_BaNiO_5_ as *E* ≈ 0.25 meV, i.e., *E* ≈ 2.0 cm^−1^. The usage of the fixed ratio *D*/*J* = 0.18 seems doubtful.

Similar comments as for Ref. [11] above apply also for Ref. [9] concerning interpretation of the average gap Δ_av_ and the directional properties of the gaps Δ_*p*_ implied by the subscripts *p* = *a*, *b*, and *c* pertaining to the crystallographic axes. Note that orientation of the axes adopted in SH, Eq. (1), bears on interpretations of the (supposedly) anisotropic nature of the Haldane gaps. The authors [9] state that Y_2_BaNiO_5_ has a body-centered orthorhombic structure, space group *Immm*, with the lattice parameters given as: *a* = 3.7648 Å, *b* = 5.7550 Å, and *c* = 11.324 Å at *T* = 10 K. Since ‘the three principal orthorhombic axes’ were defined as ‘along the edges of the orthorhombic unit cell’ an additional question arises whether the axes (*a*, *b*, *c*) coincide with the symmetry axes at the Ni^2+^ site. Judging from the crystal structure, see, e.g., Fig. 1 in Ref. [10], it seems not to be the case if the site symmetry is orthorhombic kind I, it is valid only if it is orthorhombic kind II; in the full paper, these questions will be dealt in detail.

Sakaguchi et al. [8]: YBNO sample: composite of 11 oriented small SCs and P; INS: 7–80 K; SH: equivalent to Eq. (1) with the ZFS named ‘SIA’ and *D*, *E* as ‘the single site anisotropy parameters’. The axes adopted: the *z* axis ∥ to the chain axis ≡ the *a* axis in the scattering plane; the *y* axis also in the scattering plane; the *x* axis ⊥ to the scattering plane. The derived values of the ZFSPs *D* and *E* (named ‘SIA’ [8]) for the single Ni^2+^ (*S* = 1) ion were obtained in the following way. The INS data [8] indicated the splitting of the excited triplet state to Δ_⊥_ and Δ_∥_ given by $\Updelta_{ \bot } - \Updelta_{\parallel } \approx 1.98\left| {D/k_{B} } \right|$. The authors state that although the result of [12] was derived for *D* ≥ 0 case, since the sign of *D* only determines the sequence of Δ_⊥_ and Δ_∥_, it is also applicable for negative *D*. The experimental results obtained from fitting INS peak positions at *T* = 7 K [8]: $\bar{\Updelta }_{ \bot } - \Updelta_{\parallel } = (\Updelta_{a} + \Updelta_{c} )/2 - \Updelta_{\parallel } \approx 1.55\,\,{\text{meV}}$ yielded $\left| {D/k_{B} } \right| \approx 0.78\,\,{\text{meV}} .$ Relating the anisotropy in the transverse fluctuations to $\Updelta_{c} - \Updelta_{b} \approx 1.97 \times 2\left| E \right| \approx 3.94\left| E \right| \approx 0.9\,\,{\text{meV}}$ yielded $\left| E \right| \approx 0.23\,\,{\text{meV}}$. The average gap was calculated by the relation: $(\Updelta_{\parallel } + \Updelta_{x} + \Updelta_{y} )/3,$ whereas the subscripts *x* and *y* were not defined earlier in Ref. [8]. Reanalysis of the raw INS data [8] is necessary to extract more reliable values of *D* and *E* using the theoretical expressions [13]. For comparison, the data extracted from Ref. [8] are in (cm^−1^): Δ_*a*_ = 60.5, Δ_*b*_ = 69.4, Δ_*c*_ = 76.6, Δ_av_ = 68.6, and *D* ≈ |6.29|, *E* ≈ |1.85| yielding |*E*/*D*| = 0.295. Note that in Abstract in Ref. [8], the results with fully rhombic ratio |*E*/*D*| = 1/3 were also provided: |*D*/*J*| = 0.03, |*E*/*J*| ~ 0.01, *J*/*k*_*B*_ = −24.1 meV yielding |*D*| ≈ 5.83, |*E*| ≈ 1.94 in cm^−1^.

Imanaka et al. [28]: YBNO sample: SC; HMF–EMR: pulsed magnetic field *B* up to 100 T; frequency ν = 118.8–418.6 μm; *T* = 1.6–175 K; SH: no explicit SH given but the parameters ‘*D* and *E*’ used in equations and in text named as, e.g., ‘the zero-field splitting of the triplet state for the single-ion anisotropy of the Ni^2+^ such as *D* and *E* term’ and ‘the single-ion anisotropic energy D*’. From the measured transition energies [28] between the singlet ground states and the triplet excited state (their Fig. 4), *D* was estimated as *D* = −5.81 cm^−1^. Importantly, Eqs. (1) to (3) used for curve fitting of data in Fig. 4 of Ref. [28] resemble the solutions for energies of any spin *S* = 1 system (see below), expressed in terms of the conventional ZFSPs *D* and *E* [23–25], only shifted by Δ, i.e., the Haldane gap energy. The estimated values are [28]: Δ = 42.1 and |*D*| = 5.81 (cm^−1^) with *E* = 0 assumed. Several problems may be noted: (1) the estimated gap Δ is about half of the value obtained from INS [8, 9, 11], (2) there is an ambiguity in sign of *D*—only one branch was observed, which did not allow for determination of the sign of *D*, (3) the meaning of *D*—it appears that the ZFSP *D*, ascribed explicitly in text (see above) to the ZFS of the single Ni^2+^ ion, in fact, corresponds to the ZFS within the excited triplet *S*_T_ = 1 states, i.e., *D* of Ref. [28] means the ZFS parameter D*.

The above analysis of the reported values of the ZFSPs for the single Ni^2+^ (*S* = 1) ions in YBNO reveals several doubts concerning the validity of interpretation of the raw experimental data in Refs. [8, 9, 11, 28] as well as the disparities exhibited by the ZFSP values obtained by various authors. These findings pose a dilemma: which experimental ZFSP sets may be considered as reliable? This situation calls for independent verification of both theoretical models used in studies of Haldane gap systems as well as the resulting experimental ZFSPs. The latter aspect may be dealt with the theoretical ZFSP modeling considered in Sect. 4 and applied to the pertinent data for YBNO in Sect. 5.

## Theoretical Background for Modeling of Spectroscopic Properties for Ni^2+^ Ions

### Crystal Field Analysis (CFA) and Microscopic Spin Hamiltonian (MSH) Approaches

The crystal field analysis and microscopic spin Hamiltonian package CFA/MSH [29–32] enables modeling of spectroscopic properties, and thus, to a certain extent, also magnetic properties, of transition-metal ions in crystals based on the complete diagonalization method within the whole 3*d* ^*N*^ configuration for arbitrary symmetry and axial symmetry in the case of optical and EMR data, respectively. The total Hamiltonian used in the CFA calculations is given by:

6 $$ H = H_{\text{ee}} (B,C) + H_{\text{CF}} (B_{kq} ) + H_{\text{m}} (\zeta ,M_{0} ,M_{2} ) $$

where the respective terms represent the Coulomb interactions, CF, and magnetic interactions that include, apart from the spin–orbit (SO) interaction, also the spin–other-orbit (SOO) and spin–spin (SS) interactions:

7 $$ H_{m} = H_{\text{SO}} (\zeta ) + H_{\text{SOO}} (M_{0} ,M_{2} ) + H_{\text{SS}} (M_{0} ,M_{2} ). $$

Computational details, the explicit forms of the terms in Eqs. (6) and (7), and background theory may be found in Refs. [29–32]. The Hamiltonian matrices obtained in this way are the functions of the free-ion Racah parameters *B* and *C*, the CF parameters *B*_*kq*_ (in the Wybourne notation [33, 34]), the SO constant $\zeta$, and the SS and SOO parameters *M*_0_, *M*_2_. Provided the values of these microscopic parameters are available, diagonalization of the full Hamiltonian matrices yields the energy levels and eigenvectors.

For Ni^2+^(3*d* ^8^) ions, the ground state is the orbital singlet state (^3^ *A*_2_) split by combined action of the CF and SO/SS/SOO interactions, yielding the single-ion ZFS, i.e., the three lowest energy levels arising from the ground state ^3^ *A*_2_. These ‘physical’ ZFS energy levels and the corresponding eigenvectors, which include admixtures of the excited states arising from various ^2*S* + 1^L multiplets, obtained using the package CFA/MSH [29–32] are used for the model calculations of the ZFSPs. Since the module MSH within the package CFA is applicable only for axial symmetry, we have developed suitable equations to extract the ZFSPs from the ‘physical’ ZFS energy levels (see “Appendix”). Below, we denote this approach as ‘CFA + MSH’ to distinguish it from the direct CFA/MSH approach [29–32].

For ZFSP modeling, we utilize also the approximate MSH formulas derived for Ni^2+^(3*d* ^8^) ions at orthorhombic symmetry sites by Chen and Zhao [35] within the framework of the crystal field theory using perturbation theory (PT). This approach is denoted below as PT/MSH. The rhombic CF Hamiltonian was defined in Ref. [35] as:

8 $$ H_{\text{CF}} = \sum\limits_{i} {\left[ \begin{gathered} 2\sqrt {\tfrac{4\pi }{5}} A_{20} Z_{20} (\theta_{i} ,\phi_{i} ) + 2\sqrt 3 \sqrt {\tfrac{4\pi }{5}} A_{22} Z_{22}^{c} (\theta_{i} ,\phi_{i} ) \hfill \\ + \tfrac{1}{2}\sqrt {4\pi } A_{40} Z_{40} (\theta_{i} ,\phi_{i} ) + \sqrt 5 \sqrt {4\pi } A_{42} Z_{42}^{c} (\theta_{i} ,\phi_{i} ) + \tfrac{{\sqrt {35} }}{2}\sqrt {4\pi } A_{44} Z_{44}^{c} (\theta_{i} ,\phi_{i} ) \hfill \\ \end{gathered} \right]} . $$

To enable comparison of the CFP values, we have derived the conversions relation between the CFPs $A_{kq}$ in Eq. (8) and the Wybourne CFPs $B_{kq}$ in Eq. (6):

9 $$ B_{k0} = b_{0}^{k} \cdot A_{k0} ,\,\quad \text{Re} B_{kq} = b_{q}^{k} \cdot A_{kq} \,\,{\text{for }}q = 2,4 $$

where the conversions factors $b_{q}^{k}$ are: $2,\,\sqrt 6 ,\,{3 \mathord{\left/ {\vphantom {3 2}} \right. \kern-0pt} 2},\,{{3\sqrt {10} } \mathord{\left/ {\vphantom {{3\sqrt {10} } 2}} \right. \kern-0pt} 2}$ and ${{3\sqrt {70} } \mathord{\left/ {\vphantom {{3\sqrt {70} } 2}} \right. \kern-0pt} 2}$ for (*k*, *q*): (2,0), (2,2), (4,0), (4,2), and (4,4), respectively.

The high-order perturbation formulas for 3*d* ^8^ ions in the orthorhombic CF [35] relate the ZFSPs (*D*, *E*) with the microscopic parameters: the SO constant $\zeta$, the zero-order energy levels of 3*d* ^8^ ions in the cubic CF: $D_{i} (i = 1\;{\text{to}}\;10),$ and the conventional [29] CFPs $(D_{s} ,D_{t} ,D_{\xi } ,D_{\eta } )$. Thus, the perturbation treatment used in the PT/MSH approach involves the intermediate step of separating the CF terms into the cubic and lower symmetry parts. Note that the relations between the conventional CFPs provided in Ref. [35] include an incorrect relation (probably a misprint only): $D_{s} = \frac{2}{7}A_{22}$, it should read $D_{s} = \frac{2}{7}A_{20}$. For PT/MSH modeling of the ZFSPs (*D*, *E*), a computer program has been worked out independently by two co-authors. Several numerical tests have been carried out, thus, indicating reliability of both programs. The inconsistencies concerning the sign convention for the SO constant $\zeta$ identified in Ref. [35] and their implications are to be discussed elsewhere.

### Modeling of CFPs: Point-Charge Model (PCM) vs. Superposition Model (SPM) Analysis

Reliable values of CFPs are required for the CFA/MSH or CFA + MSH and PT/MSH modeling. However, no suitable experimental data on CFPs are available for Haldane gap systems, including YBNO. Hence, we resort to the PCM utilized in Ref. [35] and the more reliable SPM analysis (for references, see, e.g., Ref. [36]). In the point-charge and dipole model (the electric dipole moment $\mu = 0$ for O^2−^ [37]) for 3*d* ^8^ ion at *D*_2h_ symmetry site applicable to Ni^2+^ in YBNO [10], the CFPs *A*_*kq*_ are given by [35]:

10 $$ \begin{aligned} A_{20} & = - (eq)\left\langle {r^{2} } \right\rangle \left[ {\frac{1}{{R_{ \bot }^{3} }} - \frac{1}{{R_{\parallel }^{3} }}} \right],\quad A_{22} = \left( {eq} \right)\left\langle {r^{2} } \right\rangle \frac{\cos \phi }{{R_{ \bot }^{3} }},\,\;A_{40} = \frac{1}{3}(eq)\left\langle {r^{4} } \right\rangle \left[ {\frac{3}{{R_{ \bot }^{5} }} - \frac{4}{{R_{\parallel }^{5} }}} \right] \\ A_{42} & = - \frac{1}{3}(eq)\left\langle {r^{4} } \right\rangle \frac{\cos \phi }{{R_{ \bot }^{5} }},\quad A_{44} = \frac{1}{3}(eq)\left\langle {r^{4} } \right\rangle \frac{\cos 2\phi }{{R_{ \bot }^{5} }} \\ \end{aligned} $$

where $q = 2e$ for O^2−^, $R_{\parallel }$ denotes the M–L bond distance along the chosen *z* axis, $R_{ \bot }$ is the M–L bond distance in the plane which is perpendicular to the *z* axis, $\phi$ is the bond angle measuring the orthorhombic distortion. The PCM [35] predicts the cubic CFP *Dq* (note that the notation $D_{q}$ used in Ref. [35] is inappropriate [29]) as:

11 $$ Dq = - \frac{{(eq)\left\langle {r^{4} } \right\rangle }}{{6R_{0}^{5} }}, $$

where $R_{0} = {{\left( {2R_{\parallel } + 4R_{ \bot } } \right)} \mathord{\left/ {\vphantom {{\left( {2R_{\parallel } + 4R_{ \bot } } \right)} 6}} \right. \kern-0pt} 6}$. The free-ion parameters for Ni^2+^ are adopted as: $B_{0} = 1,208\;{\text{cm}}^{ - 1}$, $C_{0} = 4,459\;{\text{cm}}^{ - 1}$, $\zeta_{0} = - 636\;{\text{cm}}^{ - 1}$, $\left\langle {r^{2} } \right\rangle_{0} = 1.8904\;{\text{at}} .\,{\text{units}}$, $\left\langle {r^{4} } \right\rangle_{0} = 13.4043\;\,{\text{at}} . {\text{units}}$, whereas their values reduced in crystals as: $B = N^{4} B_{0} ,\,\;C = N^{4} C_{0} ,\,\;\zeta = N^{2} \zeta_{0} ,\,\;\left\langle {r^{n} } \right\rangle = N^{2} \left\langle {r^{n} } \right\rangle_{0} ,$ where $N = \sqrt k = 0.943$ for Ni^2+^ in MgO [37].

In the SPM (see, e.g., [38]) the CFPs in the Wybourne notation can be expressed as:

12 $$ B_{kq} = \sum\limits_{L} {\bar{B}_{k} (R_{L} )K_{k}^{q} (\theta_{L} ,\phi_{L} ){{\alpha_{k,0} } \mathord{\left/ {\vphantom {{\alpha_{k,0} } {\alpha_{k,q} }}} \right. \kern-0pt} {\alpha_{k,q} }}} , $$

where $\alpha_{k,q}$ are the conversion factors [23, 38], the coordination factors $K_{k}^{q} (\theta_{\text{L}} ,\phi_{\text{L}} )$ are functions of the position angles $\theta_{\text{L}}$ and $\phi_{\text{L}}$ of ligands, whereas the intrinsic parameters $\bar{B}_{k} \left( {R_{\text{L}} } \right)$ expressed in the Wybourne notation are assumed to obey the power law:

13 $$ \bar{B}_{k} (R_{\text{L}} ) = \bar{B}_{k} \left( {R_{0} } \right)\left( {\frac{{R_{0} }}{{R_{\text{L}} }}} \right)^{{t_{k} }} , $$

where *R*_0_ and *R*_L_ are the reference distance and the distance of the *i*th ligand, respectively. The power law exponents *t*_*k*_ are treated as adjustable parameters. For SPM/CFP modeling, we employ two versions of computer program SPM worked out independently by two co-authors, which have so far been thoroughly tested by several applications to various ion-host systems (for references, see, e.g., [38, 39]), thus, indicating reliability of both programs.

## Results for Ni^2+^ Ions in Y_2_BaNiO_5_

### Crystal Structure of Y_2_BaNiO_5_

**Fig. 1:**
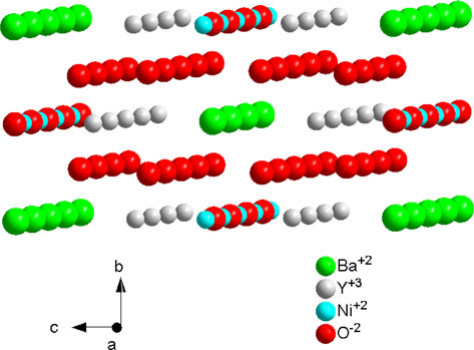
Structure of Y_2_BaNiO_5_, with the Ni–O–Ni chains along the* a axis (color online)*

**Fig. 2:**
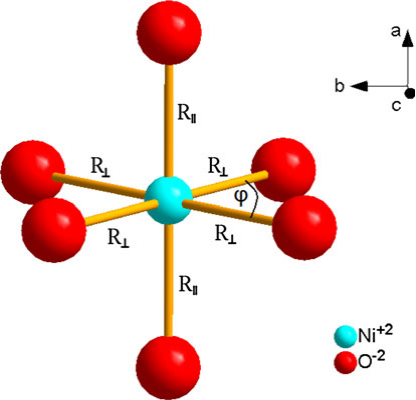
Local site symmetry of the NiO_6_ complex in Y_2_BaNiO_5_ *(color online)*

The structure of Y_2_BaNiO_5_ (YBNO) crystal, with the orthorhombic space group *Immm* (No. 71) at room temperature [40–44], is visualized in Fig. 1 using the unit cell parameters and atomic positions from Ref. [40]. The Ni^2+^ ions are located at six crystallographically equivalent sites having the *D*_2h_ symmetry. Each nickel metal (M) site is coordinated by six oxygen ligands (L), whereas the surrounding ML_6_ complex represents an octahedron with orthorhombic distortion (see, Fig. 2). All Ni^2+^ sites are magnetically equivalent and the local symmetry-adapted axis systems (SAAS: *x*, *y*, *z*) are co-parallel to the crystallographic axis system (CAS: *a*, *b*, *c*). However, the assignment of the ‘labels’ (*x*, *y*, *z*) to the axes (*a*, *b*, *c*) is arbitrary. Importantly, the values of (*D*, *E*) expressed in some of the axis systems (*x*, *y*, *z*) may become ‘non-standard’—as defined in Ref. [45] (see also [46, 47]), i.e., yielding |*E*/*D*| ≥ 1/3. The aspects of the orthorhombic standardization [45–47] pertinent for the ZFSP modeling are discussed in Sect. 5.2.

However, the existing three twofold symmetry axes (*C*_2_) are parallel to the crystallographic (*a*, *b*, *c*) axes, whereas the horizontal mirror plane (σ_h_) is perpendicular to the *a* axis (see, Fig. 2). The structural parameters for the NiO_6_ (*D*_2h_) complex in YBNO calculated from the crystallographic data [40–44] are listed in Table 1.

**Table 1:** The structural parameters for the NiO_6_ (*D*_2h_) complex in Y_2_BaNiO_5_

References:	[40]	[41]	[42]	[43]	[44]	Average
Set #:	#1	#2	#3	#4	#5	#Ave
$R_{\parallel } \text{(nm)}$	0.18851	0.18805	0.18800	0.18811	0.18796	0.18813(1)
$R_{ \bot } \text{(nm)}$	0.21854	0.21816	0.21889	0.21856	0.21783	0.21840(18)
$\phi \;(^\circ )$	78.502	78.971	78.399	78.629	78.413	78.583(105)

### Modeling of CFPs and ZFSPs

The PCM/CFP and SPM/CFP calculations utilize the structural parameters in Table 1. The calculations were carried out for all parameter sets #1–#5 in Table 1. However, due to small differences between the results, only the average values, $\bar{x}$, and their standard deviations of the mean, $u(\bar{x})$, are provided below for a given set of generic quantities, *x* = {*x*_*i*_}, defined as:

14 $$ u(\bar{x}) = \sqrt {{{\sum\limits_{i = 1}^{n} {(\bar{x} - x_{i} )^{2} } } \mathord{\left/ {\vphantom {{\sum\limits_{i = 1}^{n} {(\bar{x} - x_{i} )^{2} } } {n(n - 1)}}} \right. \kern-0pt} {n(n - 1)}}} , $$

where *n* is number of sets. The average values $\bar{x} (u(\bar{x}))$ are obtained as: the reference distance *R*_0_ = 0.2083(1) nm and the cubic CFP *Dq* = −921(3) cm^−1^. The CFPs used as input for the PT/MSH as well as CFA + MSH (see “Appendix”) calculations and subsequently the ZFSPs are obtained for Ni^2+^ in YBNO using the following procedure.

In the first stage, the CFPs $$ A_{kq} $$ in Eq. (10) are calculated using PCM for the sets #1–#5 in Table 1, yielding the average CFPs (in cm^−1^): $$ A_{20} = 5,918(31), $$ $$ A_{22} = 2,075(18), $$ $$ A_{40} = 16,620(41), $$ $$ A_{42} = - 288(3), $$ $$ A_{44} = - 1,340(6) $$. Using these CFPs as input for the PT/MSH approach yields the average ZFSPs (in cm^−1^): *D* = −5.27(6) and *E* = 1.14(2). Next, the individual PCM/CFPs $$ A_{kq} $$ (#1–#5) are converted to the CFPs $$ B_{kq} $$ using Eq. (9), yielding the average CFPs (in cm^−1^): *B*_20_ = 11,836(61), $$ \text{Re} B_{22} = 5,083(45), $$ *B*_40_ = 24,930(63), $$ \text{Re} B_{42} = - 1,365(13), $$ $$ \text{Re} B_{44} = - 16,818(81). $$ Then, using these PCM/CFPs as input for the CFA calculations (with the same free-ion parameters as used for the PT/MSH approach) yields the first three spin energy levels (in cm^−1^): *E*_1_ = 0, i.e., the ground spin state within the ^3^ *A*_2_ multiplet, the excited ^3^ *A*_2_ states: *E*_2_ = 0.342(53), *E*_3_ = 6.529(52). Subsequently, using these *E*_*i*_ values for CFA + MSH calculations yield the ZFSPs (#1–#5) from which the average ZFSPs are obtained as (in cm^−1^): *D* = −6.36(5) and *E* = 0.17(3). Note that the next energy level arising from a higher-lying multiplet is obtained at *E*_4_ = 10,444(40) cm^−1^, which is high enough to verify that it is well separated from the ground orbital singlet states, which is the necessary condition for a valid application of the MSH approach within the ^3^ *A*_2_ multiplet. The actual composition of the eigenvector of the level *E*_4_, i.e., the admixtures coefficients of the excited states arising from various ^2*S* + 1^L multiplets, may be determined from analysis of the CFA outputs.

It turns out that the PCM/CFPs used as input for the CFA + MSH and PT/MSH calculations yield similar values of the axial ZFSP *D* but completely different values for *E*. The PT/MSH approach yields a larger rhombicity ratio *E*/*D* = −0.215(2), which is still much smaller than that for the fully rhombic case |*E*/*D*| = 1/3. Importantly, the more reliable CFA + MSH approach yields very small *E*/*D* = −0.027(4). In view of this disparity, in the second stage, we calculate independently the SPM/CFPs $B_{kq}$ (#1–#5) adopting tentatively the values of the SPM parameters arising from the PCM calculations, i.e., (in cm^−1^): *t*_2_ = 3; *t*_4_ = 5; $\bar{B}_{2} (R_{0} )$ = 12,366 cm^−1^; $\bar{B}_{4} (R_{0} )$ = 5,496 cm^−1^; *R*_0_ = 0.2085 nm. The average CFPs obtained in this way (in cm^−1^)—$B_{20}$ = 12,717(292), $\text{Re} B_{22}$ = 5,459(119), $B_{40}$ = 24,937(67), $\text{Re} B_{42}$ = −1,365(13), $\text{Re} B_{44}$ = −16,817(82)—serve as input for the CFA + MSH calculations yielding the average ZFSPs (in cm^−1^): *D* = −5.30(6) and *E* = 1.13(1); *E*/*D* = −0.214(2). Comparison of the SPM and PCM calculated CFPs indicates differences only in the second-rank CFPs, while the fourth-rank CFPs are nearly the same.

**Fig. 3:**
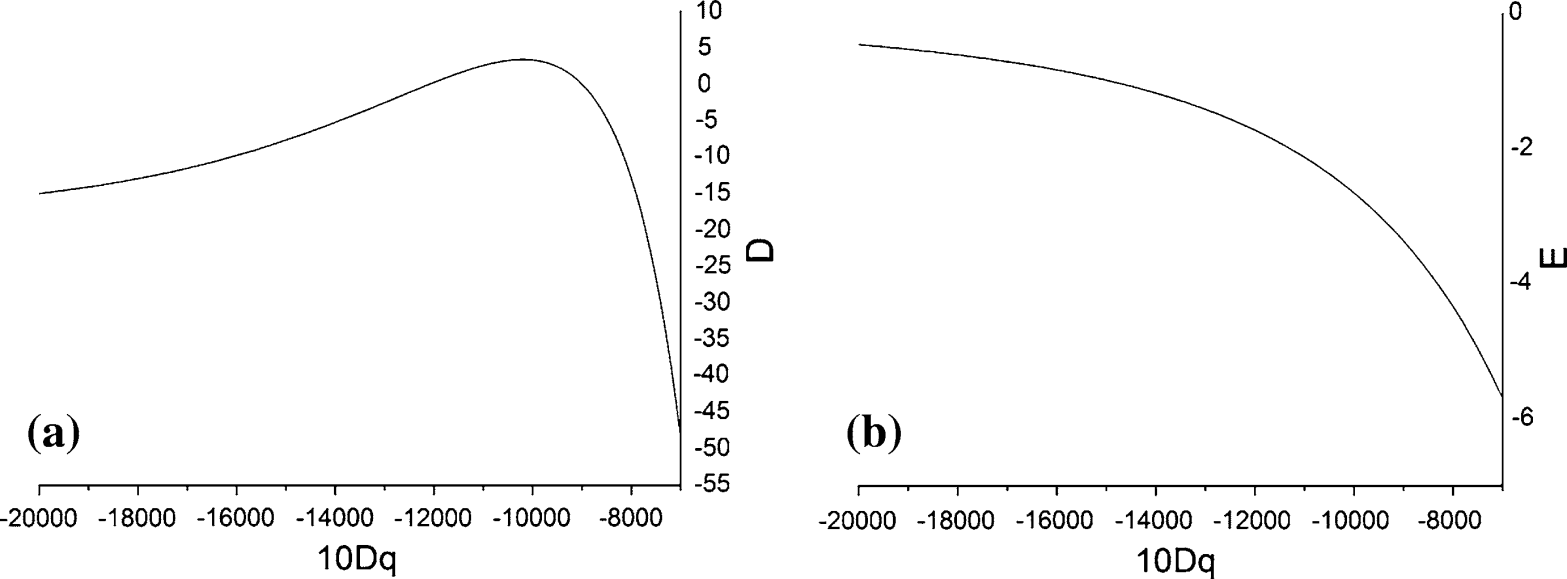
The dependence of the ZFSPs obtained from PT/MSH on the cubic CF splitting *10Dq*: **a** *D* and **b** *E*

To clarify the origin of the differences between the results from the PT/MSH and CFA + MSH approaches, we consider the dependence of the ZFSPs (*D*, *E*) on the value of the cubic CFP *Dq* inherently used in the PT/MSH approach [35]. Since *Dq* depends significantly on the reference distance *R*_0_, we have tested the effect of variation *R*_0_ on the ZFSPs obtained from PT/MSH using only one structural dataset #1 for Y_2_BaNiO_5_ [40] with *R*_0_ in the range of values approximately between those for $R_{\parallel }$ and $R_{ \bot }$. The dependence of the ZFSPs (*D*, *E*) from the PT/MSH on the value of the cubic CF splitting 10*Dq* is presented in Fig. 3, whereas the numerical results used for subsequent analysis are listed in Table 2.

**Table 2:** The effect of the cubic CF splitting [*Dq* (cm^−1^)] on the ZFSPs [*D*, *E* (cm^−1^)] for *R*_0_ (nm) ranging from about $R_{\parallel }$ to $R_{ \bot }$

*R*_0_	Si	0.180	0.185	0.190	0.195	0.200	0.205	0.210	0.215	0.220
*Dq*		−1,911	−1,667	−1,459	−1,281	−1,129	−998	−884	−786	−701
*D*	S1	−**14.2**	−**11.0**	−**6.70**	−2.00	1.99	3.30	−1.16	−**16.0**	−**48.0**
*E*		−**0.519**	−**0.750**	−**1.06**	−1.47	−2.00	−2.67	−3.50	−**4.51**	−**5.70**
*E*/*D*		**0.0366**	**0.0684**	**0.158**	0.736	−1.01	−0.811	3.02	**0.282**	**0.119**
*D*	S2	7.88	6.61	4.94	**3.21**	2.01	2.36	**5.83**	14.8	32.6
*E*		6.84	5.11	2.82	**0.264**	−1.99	−2.98	−**1.17**	5.75	21.2
*E*/*D*		0.868	0.773	0.571	**0.082**	−0.992	−1.26	−**0.201**	0.389	0.650
*D*	S4	6.32	4.36	1.76	−1.21	−**4.00**	−**5.66**	−4.67	1.24	15.5
*E*		7.36	5.86	3.88	1.74	**0.008**	−**0.311**	2.33	10.3	26.8
*E*/*D*		1.16	1.34	2.21	−1.44	−**0.002**	**0.055**	−0.499	8.25	1.74

It appears that within the range of 10*Dq* values considered, the resulting ZFSPs (*D*, *E*) become non-standard (i.e., |*E*/*D*| ≥ 1/3) for specific *Dq* values as listed in the set *S*_1_ in Table 2. These sets are expressed in disparate nominal axis systems, and hence, cannot be directly compared (for details and references, see, [45–47]). Consequently, applying suitable orthorhombic standardization transformations [45–47]—in this case S2: (*x*, *y*, *z*) → (*x*, *z*, −*y*) and S4: (*x*, *y*, *z*) → (*z*, *x*, *y*)—the corresponding standardized (*D*, *E*) values are obtained as listed in the sets S2 and S4. The standard (*D*, *E*) values are indicated in bold in Table 2.

Major outcome arising from analysis of Table 2 and Fig. 3 is that the reason the differences between the CFPs resulting from the PT/MSH approach and those from the CFA + MSH approach is that the former CFPs, unlike the latter, depend strongly on the cubic CFP *10Dq*. One also observes that for the values of *Dq* < 800 cm^−1^, the ZFSP *D* very abruptly decreases (while the absolute value increases) with *Dq*. These features reflect the approximate nature of the PT/MSH approach and constitute strong drawbacks of this approach. Fortunately, the CFA + MSH approach does not involve the intermediate step of separating the CF terms into the cubic and lower symmetry parts, inherent in the perturbation treatment used in the PT/MSH approach, and hence, this approach is not affected by the drawbacks in question. In addition, the above analysis indicates that it is possible to choose the value of *R*_0_ (thus also the value of *Dq*) in such a way that the ZFSPs resulting from both PT/MSH and CFA + MSH approaches may be similar. This makes the ZFSPs obtained from the PT/MSH approach more ambiguous.

To solve the problems encountered in the preliminary calculations presented above, several steps have to be taken in the-full scale calculations. Possible improvements include, e.g., (1) independent optical spectroscopy data on the CF energy levels and CFPs, especially the value *Dq*, should be searched from literature for comparison, (2) more reliable values of the SPM parameters, than those arising from the PCM calculations used tentatively at present, obtained from experimental data on structurally similar crystals using other theoretical or semi-empirical methods, (3) consideration of the role of more distant coordination spheres around the metal ion.

## Summary of Major Findings and Conclusions

The framework for modeling spectroscopic properties of Ni^2+^ ions in the Haldane gap systems has been worked out. Two complementary approaches are utilized, i.e., the PCM and SPM, to estimate the CFPs based on the knowledge of the crystallographic data. The CFPs serve as input for the PT expressions and the CFA package for MSH modeling of the ZFSPs *D* and *E* for Ni^2+^ ions at orthorhombic symmetry sites. The advantages of such modeling include (1) prediction of measurable parameters, (2) verification of experimental spectroscopic and structural data concerning the site symmetry and symmetry axes, (3) correlation of EMR spectroscopy and magnetic data. Extension of the modeling to include also the Zeeman factors *g*_*ij*_ is planned.

Initial applications for Ni^2+^ ions in Y_2_BaNiO_5_ presented here provide the proof of the method, i.e., the calculations show the predictive capabilities of the theoretical modeling approaches used. The results indicate that the cubic CFP (10*Dq*) depends strongly on the reference distance *R*_0_ used in the PCM/CFPs and PT/MSH calculations. This constitutes a serious limitation, which fortunately is not applicable to the calculations utilizing the SPM/CFPs and CFA + MSH approach. In general, the PT/MSH and CFA + MSH approaches are capable of predicting reasonable values of the orthorhombic ZFSPs *D* and *E* for single Ni^2+^ ions in crystals. Both positive and negative signs of *D* and values in a wide range may be obtained depending on the structural input parameters. The CFA + MSH approach provides more reliable values of *D* and *E* in terms of the estimated ZFS energy levels.

It turns out that the rhombicity ratios |*E*/*D*| for the standardized (*D*, *E*) sets predicted by the modeling (see Table 2) are generally much smaller than 1/3. Hence, the maximally rhombic ratios |*E*/*D*| ≈ 1/3 reported for the single Ni^2+^ (*S* = 1) ion in YBNO [8–10] may turn out to be computer artifacts. On the other hand, the very large tetragonal-like ZFSP *D* ~ 32 cm^−1^ [11] can be explained, since the highest predicted *D* value is |*D*| = 48.0 with |*E*| = 5.70 (cm^−1^) yielding |*E*/*D*| ≈ 0.119. However, these results indicate that the rhombic ZFS term neglected in Ref. [11] should be definitely taken into account. A final solution of the dilemma how to reconcile the reported disparate *D* and *E* values for Ni^2+^ in YBNO [8–11, 28] as well as more firm conclusions on the reliability of either results require further studies.

To utilize efficiently the predictive power of the combined SPM/CFP and CFA + MSH approaches, better sets of the input parameters for the modeling are indispensable. This can be achieved by a comprehensive survey of the pertinent parameters for Ni^2+^ (*S* = 1) ions in various Haldane gap systems as well as in other structurally similar crystals. Extensive literature search is currently under progress to provide relevant spectroscopic and magnetic data. A comparative analysis of the data available in literature will be also carried out to achieve the following goals. The predicted ZFSPs *D* and *E* for Ni^2+^ ions in various Haldane gap systems may be then verified by comparison with data for well-studied structurally similar crystals. The analysis would also help to clarify the inconsistencies occurring in the Haldane gap studies. In this way, more reliable theoretical values of the single-ion ZFSPs may be compared with the more accurate experimental values.

In parallel with the comprehensive modeling of the ZFSPs for Ni^2+^ ions in various Haldane gap systems outlined above, several crucial aspects identified so far must be reconsidered. This includes, e.g., (1) validity of equations used for the average Haldane gaps for orthorhombic and axial symmetry cases, (2) directional versus non-directional properties of Haldane gaps, (3) selection rules for various transitions between the ground singlet state and the excited triplet state, (4) implications of the existing pitfalls in interpretation of experimental data, (5) reanalysis of Batista et al. [48] model of an effective Hamiltonian used for doped crystals and consideration of its implications for pure systems.

It is also worth to mention the new perspectives arising due to advantages of the novel techniques used in Haldane gap studies. The very high pulsed magnetic fields are nowadays achievable, e.g., variable-frequency HMF–EMR was performed on Y_2_BaNiO_5_ over a wide magnetic field range up to 100 T [28]. For such high *B* values, the higher-order field-dependent terms (HOFD) with higher powers in *B*, e.g., the terms of the type: ***B*** ^***2***^ ***S*** ^***2***^, ***B*** ^***3***^ ***S***, ***B*** ^***5***^ ***S*** [23, 24], may become significant in comparison with the usual linear Zeeman term ***B***.***g***.***S***, even if the associated parameters may be small. Experimental determinations of the higher-order SH parameters from detailed fittings to HMF–EMR spectra are still hampered due to the lack of suitable theoretical framework and computer fitting programs. To enable such studies, derivation of the explicit forms of the HOFD terms in generalized spin Hamiltonians is indispensable [49]. Applications of high pressure [50] in the studies of Haldane gap systems are still scarce [51]. The theoretical approaches presented here may be also very useful for modeling of spectroscopic properties of Ni^2+^ ions in the Haldane gap systems under pressure, provided that more experimental data become available. Recent development of high-pressure, high-field and multifrequency electron spin resonance system [50] promise further advances in this novel area.

Concluding, preliminary results of this study indicate that some experimental magnetic and spectroscopic data reported earlier must be reanalyzed. It is also envisaged that alternative interpretations of experimental data are plausible and may lead to re-assignment of the transitions observed in some INS and HMF–EMR studies.

## Appendix

The CFA + MSH approach outlined in text is based on the analysis of the spin *S* = 1 energy levels obtained from the package CFA [29–32] and application of the projection method between the ‘physical’ ZFS energy levels and those of the ‘effective’ SH [23, 24]. The energy matrix for the spin (*of arbitrary nature*) *S* = 1 system in orthorhombic symmetry yields the ‘effective’ ZFS energy levels *W*_*i*_ given in Refs. [15, 16]. For the magnetic field *B* = 0, *W*_*i*_ are $W_{0} = - \tfrac{2}{3}D,\;\,W_{ \pm } = + \tfrac{1}{3}D \pm E$, and can be assigned in an arbitrary way to the ‘physical’ ZFS energy levels Δ_*j*_ (*j* = 0, 1, 2) obtained from the package CFA [29–32].

The solutions for the ZFSPs (*D*, *E*) are provided for all possible combinations of assigning the *S* = 1 energy levels Δ_*j*_ obtained using the package CFA to the ZFS energy levels *W*_*i*_ are listed in Tables 3 and 4. Note that these combinations have been here selected randomly and denoted as I–VI. Such combinations of the energy levels can also be obtained by the orthorhombic standardization transformations (*S*_*i*_ = *S*_1_ to *S*_6_) [45–47] and, thus, are related to the possible choices of axes as given by the orthorhombic standardization (see, e.g., [52]). Important point is that only one solution for the ZFSPs (*D*, *E*) is standard, whereas five other solutions are non-standard. For presentation in text, the standard solutions were selected.

**Table 3:** The *S* = 1 energy levels Δ_*j*_ (*i* = 1, 2), with the ground-state energy Δ_0_ set to zero, in terms of the ZFSPs (*D*, *E*) for all possible combinations of assignment of the ‘effective’ and ‘physical’ ZFS energy levels

	I	II	III	IV	V	VI
Δ_2_	*D* + *E*	D – *E*	2*E*	−2*E*	−*D* + *E*	*−D* − *E*
Δ_1_	*D* − *E*	*D* + *E*	*−D* + *E*	*−D* − *E*	2*E*	−2*E*

**Table 4:** The inverse relations between the ZFSPs (*D*, *E*) and the *S* = 1 energy levels Δ_*j*_ (*i* = 1, 2) with Δ_0_ = 0 for all possible assignment combinations

	I	II	III	IV	V	VI
*D*	(Δ_1_ + Δ_2_)/2	(Δ_1_ + Δ_2_)/2	−Δ_1_ + Δ_2_/2	−Δ_1_ + Δ_2_/2	−Δ_2_ + Δ_1_/2	−Δ_2_ + Δ_1_/2
*E*	(Δ_2_ – Δ_1_)/2	−(Δ_2_ – Δ_1_)/2	Δ_2_/2	−Δ_2_/2	Δ_1_/2	−Δ_1_/2
$\frac{E}{D}$	$\frac{{\Updelta_{2} - \Updelta_{1} }}{{\Updelta_{1} + \Updelta_{2} }}$	$- \frac{{\Updelta_{2} - \Updelta_{1} }}{{\Updelta_{1} + \Updelta_{2} }}$	$\frac{{\Updelta_{2} }}{{ - 2\Updelta_{1} + \Updelta_{2} }}$	$\frac{{ - \Updelta_{2} }}{{ - 2\Updelta_{1} + \Updelta_{2} }}$	$\frac{{\Updelta_{1} }}{{ - 2\Updelta_{2} + \Updelta_{1} }}$	$\frac{{ - \Updelta_{1} }}{{ - 2\Updelta_{2} + \Updelta_{1} }}$
